# Driving toward Connectivity: Vehicular Visible Light Communications Receiver with Adaptive Field of View for Enhanced Noise Resilience and Mobility

**DOI:** 10.3390/s24092814

**Published:** 2024-04-28

**Authors:** Alin-Mihai Căilean, Sebastian-Andrei Avătămăniței, Cătălin Beguni

**Affiliations:** 1 Integrated Center for Research, Development and Innovation in Advanced Materials, Nanotechnologies and Distributed Systems for Fabrication and Control, Stefan cel Mare University of Suceava, 720229 Suceava, Romania; sebastian.avatamanitei@usm.ro (S.-A.A.); catalin.beguni@usm.ro (C.B.); 2 Department of Computers, Electronics and Automation, Stefan cel Mare University of Suceava, 720229 Suceava, Romania; 3 East European Border Scientific and Technological Park, 725500 Siret, Romania

**Keywords:** field of view (FoV), noise-adaptive, optical noise mitigation, visible light communication (VLC)

## Abstract

Wireless communication represents the basis for the next generation of vehicle safety systems, whereas visible light communication (VLC) is one of the most suitable technologies for this purpose. In this context, this work introduces a novel VLC receiver architecture that integrates a field-of-view (FoV) adaptation mechanism in accordance with the optical noise generated by the sun. In order to demonstrate the benefits of this concept, a VLC prototype was experimentally tested in an infrastructure-to-vehicle (I2V) VLC configuration, which uses an LED traffic light as the transmitter. At the receiver side, an automatic FoV adaptation mechanism was designed based on a mechanical iris placed in front of a photodetector. Adjustments were made based on the values recorded by a multi-angle light sensor, built with an array of IR photodiodes covering an elevation from 0° to 30° and an azimuth from −30° to 30°. Depending on the incidence of solar light, the mechanical iris can adjust the FoV from ±1° to ±22°, taking into account both the light irradiance and the sun’s position relative to the VLC receiver. For experimental testing, two identical VLC receivers were used: one with an automatic FoV adjustment, and the other with a ±22° fixed FoV. The test results performed at a distance of 50 m, in the presence of solar irradiance reaching up to 67,000 µW/cm^2^, showed that the receiver with a fixed FoV saturated and lost the communication link most of the time, whereas the receiver with an adjustable FoV maintained an active link throughout the entire period, with a bit error rate (BER) of less than 10^−7^.

## 1. Introduction

Visible light communication (VLC) is a wireless communication technology that marks significant progress in recent years towards reliability, security, and efficiency, ensuring the transition from an emergent to a confirmed technology. VLC uses the visible light spectrum of solid-state light (SSL) devices not only for illumination, but also for simultaneous data transmission purposes [1], resulting in numerous advantages, such as energy consumption efficiency, an unpreceded bandwidth, and a never-before-imaginable pervasive spread potential throughout various domains. Based on such a high potential, VLC has been found to be appropriate for numerous applications, from high-data-rate indoor applications [2] to underwater communication [3], and more recently, for Internet of Things applications [4]. Moreover, VLC is capable of having a major impact on the development of 6G-and-beyond technologies [5,6]. In addition, intensive research efforts have demonstrated that VLC can provide high-precision positioning [7], opening the path for a multitude of new applications. Last, but not least, VLC has been found to be suitable for various environment-sensing applications [8,9,10,11]. Thus, one can see that VLC evolved from the point of being seen only as a possible technology, to the point where light sources became capable of wireless communication, range measurements, high-precision positioning, and obstacle detection, up to the point where it can provide a basis for the integrated communications and sensing (ICS) concept.

A particular use for VLC is for communication-based vehicle safety applications [12,13,14]. In this area, VLC is favored by the wide integration of SSL sources as part of vehicle lighting systems, and also as part of the transportation infrastructure, such as traffic lights, traffic signs, information display panels, and street lighting systems. Thus, the wide distribution of SSL-based sources, as well as their relatively high optical intensity, makes the vehicular use of VLC one of the most relevant and straightforward application scenarios. Moreover, the use of VLC in inter-vehicle communication is a complementary and fully compatible alternative to the traditional radio-frequency (RF) communication systems, augmenting its performance in terms of the resilience to interference and jamming attacks, ensuring lower latencies, and providing higher packet delivery ratios [12,15,16,17].

Despite the fact that this was the first domain of utilization for VLC technology, the characteristics of the outdoor channel raised numerous challenges [12]. It should be remembered here that the vehicular VLC channel is characterized as being highly dynamic and unpredictable, subject to numerous optical noise sources, and influenced by weather phenomena, such as fog, rain, and snow, which affect the passage of light, worsening the signal-to-noise ratio (SNR) [18]. Regardless of these unfriendly conditions, VLC has made impressive progress, achieving a performance that was difficult to imagine before. Thus, today’s vehicular VLC prototypes can provide a high resilience to optical interference [19,20,21], including the interference generated by multiple VLC transmitters [22]; decent data rates; and communication ranges of up to almost 200 m [23], indicating that this technology is almost ready for real-life implementation [24,25,26]. Although substantial strides have been made in advancing vehicular VLC technology, its specific challenges require further research efforts. Thus, improving the vehicular VLC resilience to noise and optimizing the compatibility with dynamic mobile conditions still remain the most important research demands [12]. From this perspective, the vehicular environment poses unique challenges, including low optical power data signals with a high potential for interference from various optical sources, inconsistent ambient lighting, and vehicle motion. Addressing these issues requires intensive efforts to develop robust noise-mitigation techniques, adaptive architectures, and eventually, efficient error-correction mechanisms. By following this path, we can unlock the full potential of VLC as a transformative technology for next-generation intelligent transportation systems.

In this context, this article proposes a new field of view (FoV) adaptive VLC receiver aimed for vehicular-communication-based safety applications. Differently from previous works, where the VLC receiver had a static FoV, the proposed concept in this work uses an automatic system, which constantly adjusts the receiver’s FoV in accordance with the intensity and location of the optical noise source relative to the photodetector. This concept aims to maintain an FoV as wide as possible, reducing it only when the intensity of the optical noise source rises to a value that endangers the reliability of the link. In order to evaluate the proposed concept, a new VLC receiver prototype was implemented and experimentally tested in outdoor sun-exposure conditions. To better emphasize the benefits of the proposed concept, the demonstrative prototype was evaluated in parallel with a similar architecture, but without the adaptive FoV option. The experimental results confirmed the benefits of the proposed concept, enabling the I2V VLC prototype to maintain a 50 m communication range for the entire day and allowing it to cope with a sunlight irradiance that rose up to 67,000 µW/cm^2^, including when the sunlight was directly incident on the VLC receiver’s lens. For comparison, the proposed concept significantly extended the connectivity compared to the situation in which the adaptive FoV was disabled. 

It should be mentioned that the authors of [27,28,29,30] have previously used the dynamic FoV concept in indoor applications, with the purpose of better coping with dense optical networks. Nevertheless, as far as we know, this is the first work that experimentally demonstrates the benefits of an adaptive FoV VLC receiver designed for vehicular VLC applications. Different from [27,28,29,30], this prototype adjusts its FoV in accordance with the location of the optical noise source relative to the VLC receiver, and also relative to its intensity. Thus, the main contributions of this work can be summarized as follows:

i. Proposing and implementing a new adaptive FoV VLC receiver concept designed for vehicular applications;
ii. Experimentally demonstrating an improved reliability for the adaptive FoV VLC receiver concept in real outdoor conditions;
iii. Paving the way for the better mitigation of interference due to multiple vehicular VLC sources;
iv. Opening the possibility of further improving the VLC receiver with more sensitive photodiodes in order to increase the communication distances;
v. Marking an important step forward in the direction of context-adaptive VLC systems.

The rest of this article is structured as follows. Section 2 provides a brief analysis, which points out some of the challenges generated by an outdoor VLC channel and presents some of the existing solutions to address these challenges. Section 3 presents the adaptive FoV VLC receiver concept developed this work. Section 4 describes the comprehensive experimental setup that was designed in order to demonstrate the viability and the benefits of the proposed concept, together with the experimental results, a discussion on the experimental results, and the future perspectives of this work. At the end, Section 5 delivers the conclusions of this article.

## 2. Enhancing the Optical Noise Resilience and Compatibility with the Mobile Conditions in Vehicular Visible Light Communication 

As described in the previous section, vehicular VLC applications are very challenging, whereas noise resilience and enhancing the compatibility with the mobile conditions are probably the most stringent issues associated with the use of VLC technology in vehicular applications [12]. The complexity of dealing with these problems becomes even more challenging because they have antagonistic characteristics, and establishing the optimal tradeoff between them is rather difficult. In fact, sacrificing the mobility of vehicular VLC systems by reducing the VLC receiver’s FoV was, for a long period, the handiest solution to improve the noise resistance [12]. Thus, in the early years, long-range vehicular VLC prototypes sometimes had FoVs as narrow as 0.4–3° [31,32]. Later on, as the design of VLC receivers improved, better tradeoff solutions were integrated. Thus, today’s vehicular VLC receivers commonly have ±20° FoVs [21,24,25,26], while sometimes reaching ±50° [25]. Nevertheless, wide FoV VLC receivers are not able to maintain an active link under sunlight conditions, or they may only be able to provide limited communication ranges. For example, the solutions presented in [23,25] were able to provide long-range communication only in indirect sunlight conditions, with the VLC receiver demonstrating an optical irradiance of up to 5000 µW/cm^2^. This demonstrates that, as the communication range increases and the SNR decreases, certain reliability issues might appear, pointing out that additional research efforts are required.

As previously mentioned, in the initial development stage, this problem was addressed mostly by reducing the VLC receiver’s FoV. This method takes advantage of VLC mandatory line-of-sight (LoS) conditions. Thus, as a VLC receiver uses a photodiode (PD) to transform light into an electrical signal, whereas a PD will generate an electrical current directly proportional to the incident light, it is obvious that less optical noise will reach the PD’s surface when the FoV is reduced. Additionally, when the FoV is reduced, part of the optical noise source is spatially isolated. This leads to lower chances of photodiode saturation and also limits the effects of light-introduced shot noise. Although rather primitive, this method is very effective at enhancing the SNR and improving the performance of VLC receivers. Nevertheless, as vehicular VLC is highly dynamic, the narrow FoV can lead to situations in which the VLC transmitter goes outside the FoV, interrupting the link. Consequently, additional optical noise-mitigation techniques are required.

Another very effective solution is represented by the use of optical filters [33,34,35]. Optical filters allow the passage of the desired wavelengths while attenuating other wavelengths. Thus, for applications that involve the use of the entire visible light spectrum, infrared (IR) reject filters are commonly used [19,23], whereas for applications that require only certain colors, narrow-band-pass optical filters are applied. In most cases, such filters have a 40 nm band-pass region [25,26], and the characteristics of the filter are selected in accordance with the VLC transmitter’s optical characteristics. The use of optical filters can eliminate more than 50% of the optical noise, significantly improving the SNR. The obvious problem with this approach is that, in a traffic scenario, the VLC receiver can only detect one wavelength of light sources, such as the red light from a vehicle’s taillight, thereby disregarding other light colors, such as the green or yellow emitted by traffic lights. Therefore, IR-reject optical filters represent an efficient tradeoff solution.

As we move forward in the VLC receiver’s architecture, the optical noise resilience can be improved with the help of an adequately designed transimpedance circuit. For example, in [19], a new logarithmic transimpedance circuit design was proposed and experimentally evaluated. The experimental results showed that such a design prevents saturation under in strong optical-noise conditions and significantly improves the dynamic range. Nevertheless, due to the fact that the design adjusts the amplification in accordance with the total light intensity received, strong optical-noise conditions can lead to insufficient amplification levels for the useful signal, and in turn, to limited communication ranges. Thus, combining this method with an adaptive FoV design can further improve its performance.

The resilience to optical interference can also be addressed with the help of improved signal-processing techniques. Both analog and digital signal-processing techniques can be used to eliminate unwanted noise components and improve the SNR [19,20,25,26,36,37]. In [20], the integration of a digital signal-processing (DSP) technique enabled the system to cope with a 12,000 lx optical interference level and almost double the communication range. Then again, the authors of [36] showed that the use of a DSP block can allow a vehicular VLC system to better deal with thermal turbulence, rain, and fog, reconfirming the benefits associated with a well-designed signal-processing plan.

In addition to hardware upgrades, the performance and resilience of vehicular VLC systems can be enhanced with the help of various modulation techniques. This aspect was pointed out by the authors of [38,39], who intensively investigated the benefits of the direct sequence spread spectrum (DSSS) technique. Their experiments showed that such a system can deal with daylight noise and can enable communication ranges of up to 40 m. The authors of [21] experimentally demonstrated that noise resilience can be improved with the help of binary phase shift keying (BPSK) modulation. This approach enabled the VLC system to better filter the noise, improving the resilience to optical interference by 25% compared to the use of the classical on—off keying (OOK) modulation. Furthermore, in combination with optical filters, the prototype was able to maintain the communication link even when exposed to optical noise levels reaching up to 65,000 µW/cm^2^. On the other hand, the authors of [40,41,42] demonstrated the enhanced noise resilience provided by the use of orthogonal frequency division multiplexing (OFDM) modulation. Again, in combination with optical filtering, it was able to cope with optical noise intensities of up to 50,000 lx [40].

In addition to the techniques mentioned above, the effect of optical interference can eventually be mitigated with the help of forward error correcting (FEC) protocols. From this perspective, the FEC protocols included in the IEEE 802.15.7 standard for visible light communication can provide outdoor VLC with a 10 dB SNR improvement [43].

In summary, the resilience of vehicular VLC systems to optical noise can be enhanced by reducing the VLC receiver’s FoV; by using optical filters; by improving the design of the transimpedance circuit; or by using various analog and digital signal-processing techniques, improved modulation techniques, and FEC protocols. Nevertheless, none of these solutions alone are able to make a VLC system fully compatible with vehicular applications. On the other hand, the combination of these techniques has the potential to provide superior vehicular VLC prototypes that are suitable for real-life applications. From this perspective, a context-adaptive VLC system seems to be the most adequate approach for developing high-performance vehicular VLC prototypes [12,37,44,45,46].

Context-adaptive VLC systems represent a promising solution, because they are able to tie together the unique advantages of VLC, such as an immunity to radio frequency interference, low latencies, and the potential for high data rates, with smart adaptations to changing conditions within the environment. In a vehicular scenario, the importance of context-adaptive VLC systems becomes even more obvious. Such flexibility is essential in coping with the variability met in real-world situations, where aspects such as the lighting conditions, obstacles, and interference levels can vary unpredictably. Therefore, by harnessing context-awareness and environment-adaptive features, VLC systems can counteract signal degradation, enhance resilience, and maximize throughput, bringing VLC technology closer to deployment in real-life applications.

## 3. Adaptive Field-of-View Visible Light Communication: Concept and Prototype Development

In light of the above, this work proposes a new vehicular VLC receiver design, in which the tradeoff between noise resilience and mobility is constantly adapted based on a context-adaptive FoV. The following section provides a description of the vehicular VLC prototype that was used to experimentally test the concept.

### 3.1. Presentation of the VLC Transmitter Used for the Experiments

**Figure 1:**
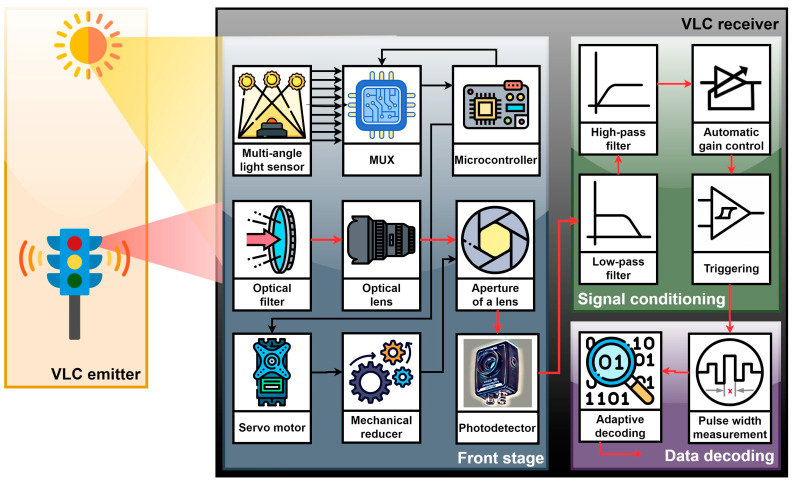
Schematic representation of the VLC prototype, emphasizing the VLC receiver’s front-end components and the adaptive field-of-view mechanism. The proposed concept used a custom-made multi-angle light sensor to determine the sun’s intensity and location relative to the VLC receiver, and based on this assessment, it adjusted the aperture of the mechanical iris in order to maintain the incident light at a value below the photodiode saturation limit.

The prototype used for the experimental testing consisted of a 20 cm diameter traffic-light VLC transmitter and a PIN photodiode-based VLC receiver. From a hardware point of view, the VLC transmitter comprised LED light sources (in this case, an LED traffic light), a digital switching driver, and a 600 MHz ARM Cortex M7 processor-based microcontroller. The traffic light served as the optical source for transmitting encoded data. For this experiment, in order to demonstrate that the concept is suitable for real-life situations, a commercial traffic light was used. It is important to emphasize that the lighting characteristics of the LED traffic light were not modified, neither by increasing the optical output nor by modifying the light distribution pattern. The traffic light LEDs were controlled by a digital switching driver, which used OOK modulation to send the data stream generated by the microcontroller. In turn, the microcontroller served as the central processing unit of the VLC transmitter, being responsible for generating and encoding the data stream, and also for building/encapsulating the data frame. The microcontroller’s software comprised algorithms for Manchester encoding, which involved converting each data bit into a pair of consecutive OOK symbols and generating frames at various data rates between 11 and 250 kb/s. The VLC transmitter used an asynchronous communication mode, meaning that the VLC transmitter and receiver were not synchronized. Asynchronous communication enabled data packets of variable lengths and flexible timing, which is suitable for applications with unpredictable data rates, variable transmission requirements, and decentralized networks. Therefore, this is an adequate option for vehicular applications. In terms of optical specifications, the LED-based traffic light had an optical irradiance of 130 µW/cm^2^ (measured at a 1 m distance), and a transmitter’s half-angle of ±15°. Overall, the VLC transmitter complied with standard optical power levels and used off-the-shelf components to ensure its cost-effectiveness, ease of implementation, and comparable results with real-life situations. Figure 1 presents the schematic block of the VLC transmitter.

### 3.2. Discussions on the VLC Receiver Architecture, Emphasizing the Structure and Importance of the Adaptive FoV Unit

The VLC receiver is the most important component in a VLC system, being responsible for detecting, processing, and extracting the information from the modulated light signals [47]. From a purpose point of view, the VLC receiver’s structure can be divided into three major blocks: the front-end optical collecting system, the signal-processing component, and the data-processing block. Figure 1 presents the architecture of the proposed concept.

The front-end optical collecting system is developed around the photosensitive element, which is responsible for the transformation of incident light power into a proportional electrical current. As in most cases, a PIN photodiode is used, due to its low response times and high sensitivity. The PIN photodiode is connected in a transimpedance circuit, providing a fair tradeoff between the bandwidth, noise, sensitivity, and dynamic range. As the output signal is dependent on the incident light power, a 2-inch optical lens is used to increase the collecting area. The optical lens focuses the incident light on the photodiode’s surface. As a result, the optical lens restricts the FoV to ±22°. In order to enhance the SNR, a 40 nm red optical filter is used to reject the upper spectrum of ambient noise.

**Figure 2:**
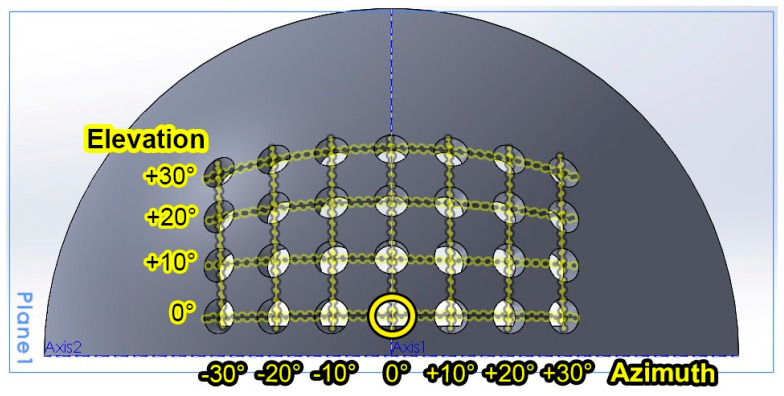
Illustration of the sun’s intensity and location-estimation unit 3D printable model, where the holes mark the position of the 28 IR photodiodes on the quarter-sphere.

In this work, the elements of novelty were introduced at the level of the front-end optical collecting system. Different from other solutions, this newly developed prototype integrated an optical-noise-sensor module coupled with the optical collecting system. The sensor module consisted of a 4 cm × 8 cm × 4 cm quarter-sphere that englobed 28 IR photodiodes, each with a ±10° FoV, distributed from −30° to 30° on the *0x* axis and from 0° to 30° on the *0y* axis, as illustrated in Figure 2. The 28 IR photodiodes were connected through a multiplexor. A microcontroller periodically read the voltage generated by the resistive network and determined the sun’s IR optical intensity. It should be clarified that the IR photodiodes were chosen in order to enable the system to respond to the sunlight instead of the light generated by the useful sources, such as the VLC transmitter. Then again, the sunlight’s IR component enabled the system to estimate the sunlight’s irradiance coming from different angles. Moreover, by comparing the irradiance from different angles, the microcontroller was able to estimate the sun’s location with respect to the VLC receiver. For this purpose, the microcontroller calculated an average of the IR light intensity received on the different angles (i.e., −30° to +30° for the azimuth and 0° to 30° for the elevation), and then, by comparing the average intensities, the position of the sun was estimated. Moreover, the use of the average values rather than individual values reduced the effect of potential measurement errors generated by imprecise 3D printing or imprecise photodiode positioning within the multi-angle light sensor. On the other hand, if no major differences were measured, the algorithm assumed that the VLC receiver was exposed to indirect sunlight. In this case, the estimated optical irradiance will be the main factor considered for the FoV adaption. From this perspective, the system was calibrated to classify the light intensity from a low level to a high level, where the light incident on the 0° axes had a higher disruptive effect at the VLC receiver level. Therefore, by correlating the sun’s location and optical intensity, the dedicated microcontroller commanded a DC motor that adjusted the aperture of a mechanical iris. The purpose of this adjustment was to ensure that the sunlight reaching the photosensitive element was always at a value that would not affect the communication parameters, where these values were established based on a preliminary set of experimental investigations.

The signal-processing component that follows had the purpose of conditioning the electrical signal furnished by the previous stage. Within this block, the signal went through a fourth-order Bessel band-pass filter. This filter rejected frequencies below 1 kHz and those above 200 kHz. The output of the filtering stage was amplified by several amplification blocks, with the final one being an automatic gain control (AGC) amplifier. The AGC stage provided complementary gain for the signals that required additional amplification, enabling the VLC receiver to be compatible with mobile applications, where the transmitter–receiver distance can vary significantly. The AGC provided an output signal with an amplitude between 0.6 V and 2 V, which was fed to a Schmitt trigger circuit. In turn, the Schmitt trigger circuit was responsible for signal regeneration. 

In the end, the digital signal generated by the Schmitt trigger circuit was processed by a 600 MHz ARM Cortex M7 processor, overclocked at 1008 MHz. The microcontroller processed the signal and extracted the data bits. More exactly, the microcontroller used interrupts to identify the rising and falling signal edges, and then it detected the received bits based on a pulse width determination. After that, the microcontroller determined the bit error ratio (BER) by comparing the received bits with the predefined bits previously stored in the memory. To investigate the performance from a hardware point of view, forward error correcting protocols were not used. Finally, the data-processing unit was interfaced with other devices through a USB connection. 

## 4. Experimental Testing Procedure, Experimental Results, and Discussions on the Benefits of the Proposed Concept

The following section presents the experimental testing procedure, with the aim of determining the benefits of the proposed concepts. After the presentation of the testing procedure, the experimental results for several experiments are provided and discussed.

### 4.1. Presentation of the Experimental Testing Procedure

This section presents the method of experimental testing, with the aim of determining the benefits of the proposed concepts by comparison with a similar prototype whose adaptive FoV characteristic was disabled. The testing procedure involved the VLC transmitter described in Section 3.1, placed at approximately 50 m from the two VLC receivers, whose basic characteristics are detailed in Section 3.2. It should be clarified that the two VLC receivers had the same hardware and software structures, and were calibrated to have the same settings (i.e., optical components, gain, filtering options, etc.), with the only difference being the adaptive FoV characteristics of one of the two VLC receivers. Additionally, it should be emphasized that a relatively long communication range was envisioned by these tests in order to take the link close to its limit. In this way, the impact of the adaptive FoV was more obvious. Otherwise, at shorter communication ranges, the higher SNR could be enough to keep the link active or to be less influenced by the sunlight.

The experimental tests were conducted over an entire day, from sunrise to sunset, in order to evaluate the variable sun’s impact on the vehicular VLC during the day. From this perspective, early in the morning and late in the afternoon, the sun’s intensity is less powerful, but the light incidence angle is lower, which has a more negative impact on the VLC receiver’s performance when it is receiving direct light exposure. In such circumstances when the VLC receiver is facing the sun, a narrow FoV could be more suitable. On the other hand, during midday, the sun’s intensity is significantly higher, reaching values that can rise up to 100,000 µW/cm^2^. Additionally, the clouds can significantly influence the sun’s impact on the VLC receiver. As the sun’s impact on the VLC receiver depends on so many aspects, it is obvious that a fixed FoV receiver is less adequate for vehicular applications.

**Figure 3:**
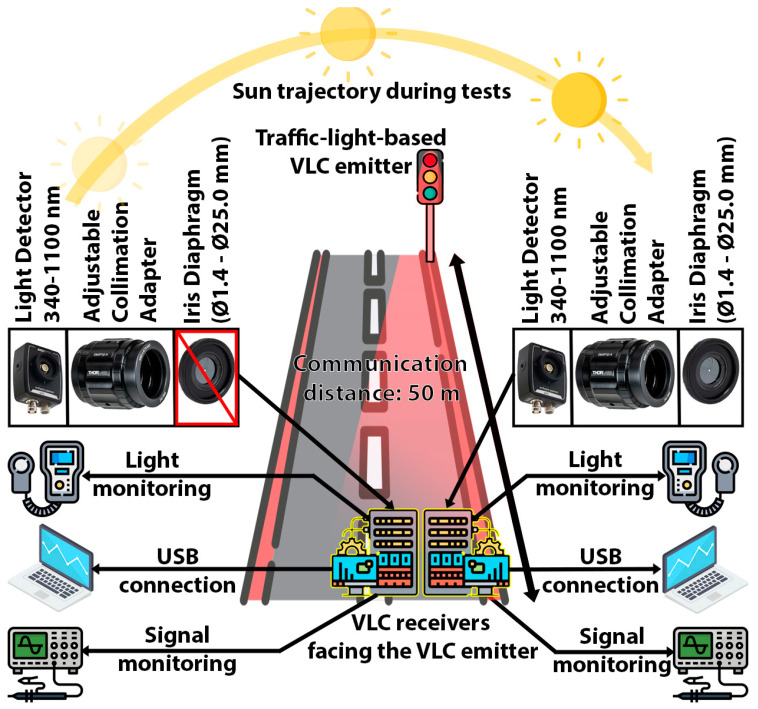
Illustration of the envisioned testing scenario: two VLC receivers, one without an adaptive FoV and one with an adaptive FoV, were monitored for an entire day. As the position of the sun varied throughout the day, it changed the impact on a 50 m VLC link between an LED-based traffic light VLC transmitter and two VLC receivers.

The schematic of the experimental testing setup is illustrated in Figure 3, exemplifying the envisioned testing scenario and the equipment layout. The projected setup assumed a vehicle approaching a traffic light. Nevertheless, depending on the moment of the day, the sun’s intensity and location, and the weather conditions (e.g., absence of clouds), the VLC receiver could experience variable optical interference levels and a variable SNR. Under certain conditions, the optical interference could be so strong that the communication link could be interrupted due to VLC receiver saturation or due to a low SNR. In such situations, a VLC receiver’s ability to adapt to the specific environment and to the specific context can be extremely important.

In summary, the aims of the following tests are provided below:

i. To investigate the system’s ability to estimate the location of the sun relative to a VLC receiver based on the IR optical power received by a custom-made multi-angle light sensor;
ii. To adjust the VLC receiver’s FoV in accordance with the incident light and the incidence angle;
iii. To experimentally determine the benefits associated with the use of the adaptive FoV VLC receiver.

### 4.2. Experimental Results

#### 4.2.1. Experimental Results for the Optical Noise Localization Component

**Figure 4:**
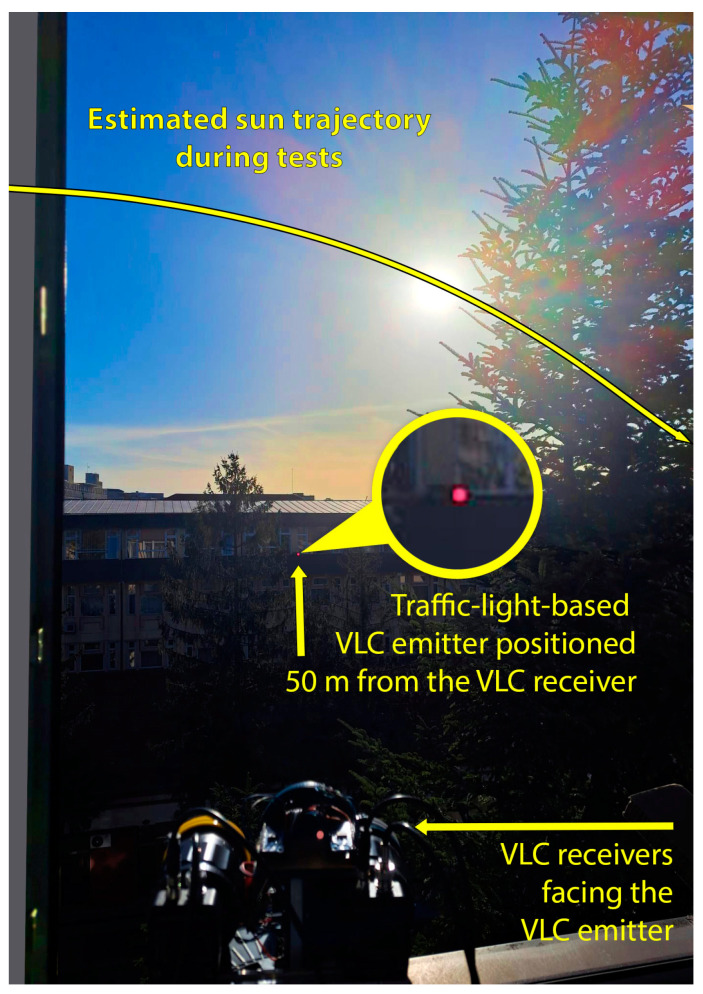
Experimental testing setup, showing the VLC transmitter, the VLC receivers, and the sun’s trajectory during the tests.

As previously mentioned, the experimental investigation was carried out for an entire day. The test setup is presented in Figure 4, showing the static experimental configuration as well as the sun’s exact trajectory during the tests. In order to avoid shadowing, we chose to place the receivers and the transmitter at the top level of two buildings located 50 m apart. As one can see, the 50 m distance made the VLC transmitter difficult to perceive.

**Figure 5:**
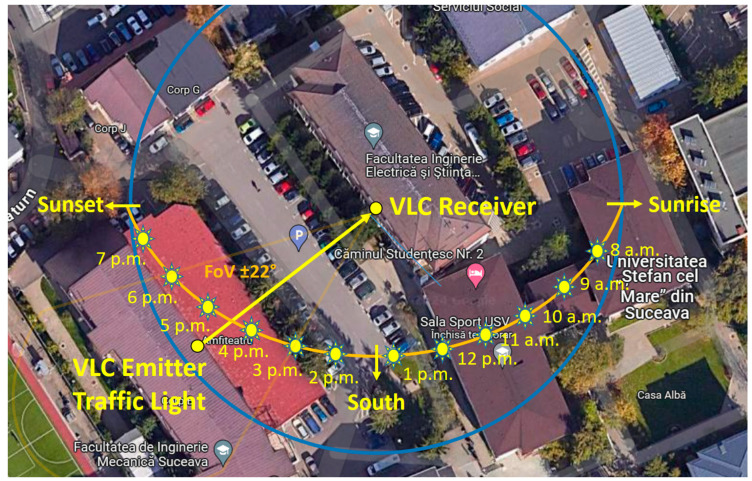
Satellite view showing the location of the VLC transmitter and VLC receivers together with the sun’s angles during the day.

The purpose of the first test was to determine if the custom-made light-detection sensor was able to determine the sun’s position relative to the VLC receiver. This aspect is very important to the next phase of the experiments, as these results will influence the adaptation of the VLC receiver’s FoV. Figure 5 shows a satellite view of the university campus where the tests were carried out. This figure shows the position of the VLC transmitter and VLC receivers, as well as the sun’s trajectory during these tests. In this case, the exact sun trajectory was established based on [48]. As one can see, in the first part of the day, the VLC receiver was not exposed to direct sunlight, but rather was only exposed to diffuse light. Later on, after 12:30 p.m., the sun began to enter the FoV of the VLC receivers. Around 4 p.m., the sun was right above the VLC transmitter, pointing directly toward the VLC receivers. After 4 p.m., the sun moved from this orientation as it approached sunset, and after 5 p.m., the sun’s influence was blocked due to a large tree (Figure 4). Additionally, the experimental tests were affected by cloudy weather from around 10:50 a.m. to 12:45 p.m.

In accordance with this setup, Table 1 summarizes the results of the experiments, which confirmed the multi-angle light sensor’s ability to estimate the sun’s relative location within a 10° limit for both axes. This was possible due to a mediation algorithm that limits the effects of certain estimation errors, and that made an assessment concerning the sun’s location based on the average of several light-intensity measurements. Additionally, it has been previously observed that the response of certain photodiodes is not in line with the response of other ones, a fact that was attributed to imprecise 3D printing and photodiode positioning within the multi-angle light sensor. Consequently, the effect of certain sensitivity discrepancies among photodiodes was optimized and calibrated with specific software algorithms. Additionally, to reduce the effects of isolated light intensity measurement errors, the decision concerning the sun’s location was made based on an averaging algorithm that considered multiple photodiode readings. It should also be mentioned that, when the multi-angle did not detect a significantly higher intensity at a certain angle, the sensor established that it was exposed to diffuse light. Under such conditions, the FoV was adapted based on the light intensity measurement, disregarding the sun’s location. Again, as the photodiodes only work with the sun’s IR component, a calibration of the light intensity measurement was required.

#### 4.2.2. Experimental Results Showing the Benefits of the Adaptive FoV VLC Receiver

**Figure 6:**
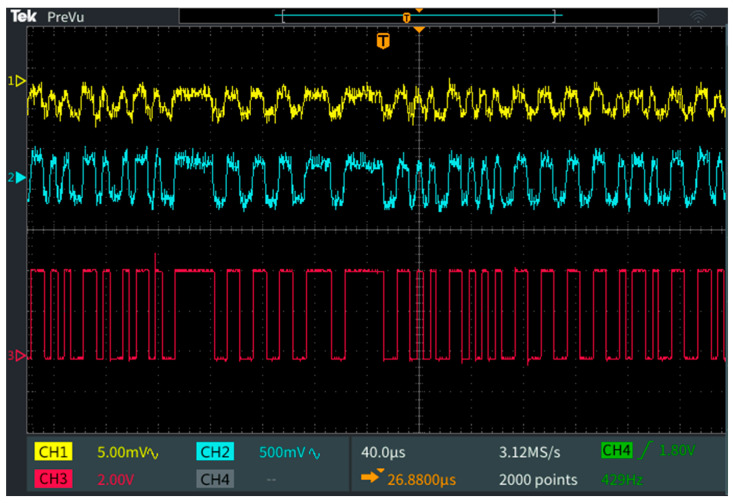
Oscilloscope screen showing the signal received by the VLC receiver.

**Figure 7:**
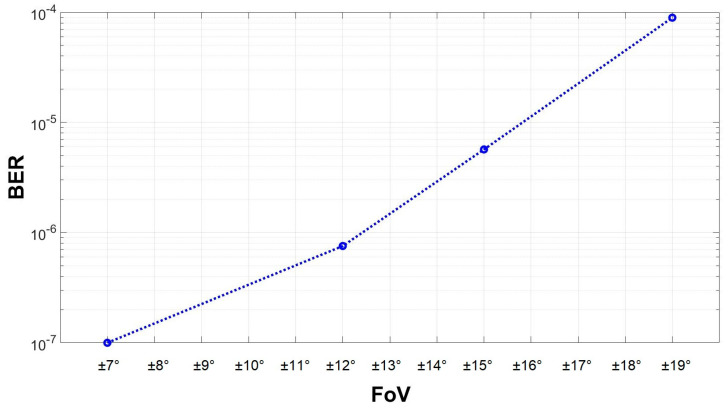
Evolution of the BER with respect to the VLC receiver’s field of view for a 50 m link under exposure to intense indirect sunlight.

The following experiments aimed to determine the benefits associated with the use of the adaptive FoV VLC receiver in terms of the BER and link operability. For improved clarity, a preliminary experimental set was made in order to better illustrate the effect of the adaptive FoV. Thus, during these tests, the VLC receiver was exposed to a sunlight irradiance of around 50,000 µW/cm^2^, and at an FoV of ±22°, the communication was lost. The FoV was decreased to ±19° in order to regain the communication, and the BER was calculated. The FoV was further decreased gradually to ±7°, while the BER was monitored. Figure 6 shows an example of the signals received by the VLC receiver, whereas Figure 7 presents the evolution of the BER with respect to the FoV. As one can see, the FoV adaptation significantly improved the reliability of the link and enhanced the BER. Thus, as the FoV was reduced from ±22°, the link was reestablished, and the BER continued to decrease from values of around 10^−4^ to values lower than 10^−7^. Consequently, these results reemphasize the significant effect that an adaptive FoV has on the VLC link performance. 

The final experimental results provided the system’s performance for an entire day, from 8 a.m. to 8 p.m. For these experiments, the BER was monitored in real time during one sunny day, from sunrise to sunset, from 8 a.m. to 8 p.m. A summary of the experimental results is provided in Table 2. To better emphasize the benefits of the adaptive mechanism, the FoV is also provided for each time interval. Additionally, Table 2 presents the sun’s irradiance, measured at the VLC receiver level with a high-precision irradiance meter (Delta Ohm 2302), as well as the sun’s location with respect to the VLC receivers. As previously shown in the experiments summarized in Figure 7, the adaptive FoV of the VLC receiver 1 was able to maintain the active link while using varying FoVs, which, in turn, led to better or worse BER results. For example, under the same conditions, a BER ranging from 10^−7^ and up to 10^−4^ could be obtained. Under such conditions, the system should consider a tradeoff between the importance of the BER and a wide FoV. In the following experiments, a low BER was considered to be a higher priority.

As one can see, in the morning between 8 a.m. and 11 a.m., when the sun was located to the left of the VLC receiver and outside the FoV, the adaptive mechanism of receiver 1 adjusted the FoV from ±22° during the first hour in the morning to ±15° when the irradiance reached values as high as 9700 µW/cm^2^. During this time, the two VLC receivers had a similar BER in the first hour, but the BER of receiver 2 increased rapidly towards 10^−4^ from 9 a.m. to 11 a.m. Over the next two hours, from about 11 a.m. to 1 p.m., some clouds cast shadows over the area, so the irradiance went up and down to a minimum of around 3640 µW/cm^2^. As a result, receiver 2 maintained the link, but at a rather high BER with respect to the adaptive receiver. From 1 p.m., the clouds were gone and the sun intensity regained its power and started increasing to a maximum of 67,000 µW/cm^2^, so that adaptive VLC receiver 1 decreased its FoV to a minimum of ±7°, preventing the degradation of the SNR and maintaining the BER at values below 10^−7^. For comparison, non-adaptive VLC receiver 2 could not be synchronized, resulting in a link loss. Starting at 3 p.m., the sun entered the FoV of both receivers. Receiver 2 continued to experience link loss, but what is important is that receiver 1 managed to have a BER of less than 10^−7^ with an FoV of ±7°. Finally, around 5 p.m., in the final phase before sunset, the shadow from a tree lowered the measured irradiance and receiver 2 regained its link, but the BER was still higher than that seen at receiver 1. Due to the lower irradiance value, the adjustable mechanism gradually widened the FoV up to ±22° at around 6 p.m., when the sun exited the FoV of the receivers, and so the BER of receiver 2 also descends to values less than 10^−7^.

### 4.3. Discussion on the Experimental Results, and Future Perspectives

The experimental evaluation of the proposed adaptive FoV VLC receiver demonstrated the benefits and advantages of this concept. As has been shown, the proposed concept has the potential to significantly improve the resilience of VLC receivers to noise while maximizing the FoV, and in turn, the systems’ mobility. Basically, establishing an optimal tradeoff between the resilience to optical noise and the compatibility with mobile conditions is an important challenge in the vehicular VLC domain [12,44]. In this context, implementing and experimentally testing such a concept represents an important step toward the deployment of this technology. It should be reemphasized that the testing setup was envisioned to take the communication link to its limits in order to better emphasize the effect of the optical interference. From this perspective, the experimental results showed that, although the non-adaptive VLC receiver was able to support a 50 m communication range, the reliability of the link was strongly affected by optical noise. Thus, as the irradiance of the optical noise increased, the BER decreased and the link was eventually lost. On the other hand, the adaptive FoV VLC receiver was able to constantly compensate for the effects of the optical noise and maintain the BER at values lower than 10^−7^. Consequently, it is obvious that the adaptive FoV architecture had a positive effect on the communication performance in terms of the BER and stability, while ensuring a decent FoV. Additionally, this architecture enabled the VLC receiver to regain its FoV once the effect of the optical noise was reduced. Therefore, enabling a VLC receiver to adapt to the environmental conditions significantly improves its performance. On the other hand, as this is still a work in progress, we can say that the FoV adaptation algorithm is still perfectible, and from this point of view, excessive FoV narrowing has been experienced. Although correlating the FoV with the noise intensity proved to be an adequate option, additional aspects should be considered. During these tests, it was observed that sometimes the multi-angle sensor was given ambiguous values at two or three adjacent photodiodes, so the position of the sun was not determined with great accuracy. In order to be on the safe side, the lowest FoV was considered in this case, to ensure that the BER was as low as possible. Nevertheless, the accuracy could be further improved based on a received DC noise signal analysis and SNR estimation, with a better hardware platform and software algorithms.

Another improvement is based on a solution integration that dynamically handles the tradeoff between the BER and a wide FoV. For example, this solution should also consider the distance between the VLC transmitter and the VLC receiver. Thus, as the distance increased, the received optical power decreased, whereas the geometric distribution led to a lower margin of error due to misalignment. Consequently, in such surroundings, reducing the FoV to mitigate the effects of optical noise and to compensate for the decreasing optical power of the data signal was perfectly justified, thus improving the SNR and avoiding the loss of link. On the other hand, when the transmitter–receiver distance decreased, the wide FoV had a lower impact on the SNR, but it facilitated the connectivity due to the LoS with the VLC emitter, increasing the mobility of the system at the same time. A dynamic adaptation of the FoV based on distance measurements could be intensively studied and variable solutions could be modeled and investigated in order for the system to move one step closer to real-life deployment. Such an approach is facilitated by the VLC systems’ ability to provide distance measurements and relative positioning as well [49,50,51]. Adaptations based on the priority of the data could also be included in a multilevel architecture for a context-adaptive system.

In light of the above, one can see that the integration of an FoV adaptation function enables the VLC system to significantly improve its resilience, mobility, and efficiency. Additionally, one can say that the integration of context-awareness and context-adaptive functions seems to be the most efficient solution to take vehicular VLC systems to the point where they are ready for deployment. Nevertheless, establishing the optimal adaptation parameters is still challenging. 

Last, but not least, it should be mentioned that the compact size of the prototype enables its integration on real cars. Moreover, the experimental testing of the system in mobile conditions with the VLC system implemented on a car are currently being performed and have provided encouraging preliminary results.

## 5. Conclusions

Further improvements to the resilience of vehicular VLC systems to optical noise and their ability to cope with various conditions represent one of the most important challenges in the development of automotive VLC systems. In this context, this article proposed a novel vehicular VLC receiver architecture that integrates an adaptive FoV mechanism. Thus, the central point of this article involved the development of an automated FoV adaptation subsystem with a mechanical iris positioned in front of a photodetector. This mechanism’s adjustments rely upon the irradiance values captured by a multi-angle light sensor, comprising an array of IR photodiodes with an elevation range of 0° to 30° and an azimuth range of −30° to 30°. Based on the sunlight intensity and the sun’s position relative to the VLC receiver, the mechanical iris can adapt the FoV from ±1° to ±22°.

In order to demonstrate the benefits of this concept, an intensive experimental evaluation was performed. In the experimental phase, two identical VLC receivers were employed—one featuring an automatic FoV adjustment and the other with a fixed FoV of ±22°. The test results revealed the benefits of a context-adaptive FoV: situated at a distance of 50 meters from an LED traffic light, under solar irradiance reaching up to 67,000 µW/cm^2^, the receiver equipped with the adjustable mechanism maintained an active link throughout the entire duration of the experiment, exhibiting a BER lower than 10^−7^. On the other hand, the receiver with a fixed FoV consistently experienced saturation and, therefore, frequent losses of connectivity. Based on these results, the conclusion drawn is that such a mechanism can improve the quality of communication in strong sunlight noise, keeping the data link active. Nevertheless, during the experimental testing, it was seen that the IR photodiode network was perfectible, providing rough estimates of the sun’s position. Even under these conditions, the system was able to satisfactorily adjust the FoV in order to maintain an active link. Additional software algorithms are needed in order to improve the detection accuracy.

In the end, one can conclude that this work provided valuable evidence concerning the benefits of an active FoV adaptation in vehicular VLC applications. As such a concept maximizes both noise resilience and mobility, it is expected that it will be further developed and eventually integrated into real-life vehicular VLC systems. As far as we know, this is the first experimental demonstration of such a concept applied in vehicular applications, opening a new path in this research domain.

## Figures and Tables

**Table 1:** Experimental results showing the sun’s angle estimation accuracy, taking into account that the line of sight between the receivers and transmitter was 232 degrees relative to north.

Moment of the Day	Sun Elevation Relative to the VLC Receiver (Real Value)	Sun Angle Relative to the VLC Receiver (Estimated Value)	Sun Azimuth Relative to the VLC Receiver (Real Value)	Sun Azimuth Relative to the VLC Receiver (Estimated Value)
8 a.m. ÷ 9 a.m.	9.75°–19.68°	Diffuse light	−137.15° ÷ −125.56°	Diffuse light
9 a.m. ÷ 10 a.m.	19.68°–29°	Diffuse light	−125.56° ÷ −112.68°	Diffuse light
10 a.m. ÷ 11 a.m.	29°–37.1°	Diffuse light	−112.68° ÷ −97.62°	Diffuse light
11 a.m. ÷ 12 p.m.	37.1°–43.16°	Diffuse light	−97.62° ÷ −79.67°	Diffuse light
12 p.m. ÷ 1 p.m.	43.16°–46.2°	Diffuse light	−79.67° ÷ −58.99°	Diffuse light
1 p.m. ÷ 2 p.m.	46.2°–45.55°	>30°	−58.99° ÷ −37.45°	>−30°
2 p.m. ÷ 2:30 p.m.	45.55°–43.45°	>30°	−37.45° ÷ −27.6°	>−30°
2:30 p.m. ÷ 3 p.m.	43.45°–41.36°	>30°	−27.6° ÷ −17.6°	−20° ÷ −30°
3 p.m. ÷ 3:30 p.m.	41.36°–37.9°	>30°	−17.6° ÷ −9.19°	−10° ÷ −20°
3:30 p.m. ÷ 4 p.m.	37.9°–34.49°	>30°	−9.19° ÷ −0.69°	<0°
4 p.m. ÷ 4:30 p.m.	34.49°–30.19°	>30°	−0.69° ÷ 6.31°	0° ÷ −10°
4:30 p.m. ÷ 5 p.m.	30.19°–25.9°	>30°	6.31° ÷ 13.52°	0° ÷ −10° −10° ÷ −20°
5 p.m. ÷ 6 p.m.	25.9°–16.32°	Diffuse light	13.52° ÷ 25.87°	Diffuse light
6 p.m. ÷ 7 p.m.	16.32°–6.31°	Diffuse light	25.87° ÷ 37.23°	Diffuse light
7 p.m. ÷ 7:45 p.m.	6.31°–0.83°	Diffuse light	37.23° ÷ 45.08°	Diffuse light

**Table 2:** Experimental results showing the BER results and the benefits of the adaptive FoV.

Hour of the Day	Sun Azimuth with Respect to VLC Receiver	Sun Elevation with Respect to VLC Receiver	Sun Irradiance Measured at the VLC Receiver [µW/cm^2^]	Adapted FoV for VLC Receiver 1	BER at VLC Receiver 1 (with Adapted FoV)	BER at VLC Receiver 2 (with Fixed FoV)
8 a.m. – 9 a.m.	−137.15° – −125.56°	9.75° – 19.68°	2780 – 4400	±22° – ±19°	<10^−7^	<10^−7^
9 a.m. – 10 a.m.	−125.56° – −112.68°	19.68° – 29°	4400 – 7090	±19° – ±15°	<10^−7^	10^−6^ – 10^−5^
10 a.m. – 11 a.m.*	−112.68° – −97.62°	29° – 37.1°	7090 – 9700	±15°	<10^−7^	10^−5^ – 10^−4^
11 a.m. – 12 p.m.*	−97.62° – −79.67°	37.1° – 43.16°	9700 – 3640	±15° – ±22°	<10^−7^	10^−4^ – <10^−6^
12 p.m. – 1 p.m.*	−79.67° – −58.99°	43.16° – 46.2°	3860 – 7100	±22° – ±15°	<10^−7^	10^−6^ – 10^−5^
1 p.m. – 2 p.m.	−58.99° – −37.45°	46.2° – 45.55°	41,700 – 43,580	±7°	<10^−7^	link loss
2 p.m. – 2:30 p.m.	−37.45° – −27.6°	45.55° – 43.45°	43,200 – 47,100	±7°	<10^−7^	link loss
2:30 p.m. – 3 p.m.	−27.6° – −17.6°	43.45° – 41.36°	47,400 – 52,000	±7°	<10^−7^	link loss
3 p.m. – 3:30 p.m.	−17.6° – −9.19°	41.36° – 37.9°	52,000 – 63,000	±7°	<10^−7^	link loss
3:30 p.m. – 4 p.m.	−9.19° – −0.69°	37.9° – 34.49°	61,300 – 67,000	±7°	<10^−7^	link loss
4 p.m. – 4:30 p.m.	−0.69° – 6.31°	34.49° – 30.19°	65,200 – 52,400	±7°	<10^−7^	link loss
4:30 p.m. – 5 p.m.	6.31° – 13.52°	30.19° – 25.9°	52,200 – 47,600	±7°	<10^−7^	link loss
5 p.m. – 5:30 p.m.**	13.52° – 21.19°	25.9° – 21.1°	47,600 – 8100	±7° – ±15°	<10^−7^	link loss – 10^−5^
5:30 p.m. – 6 p.m.	21.19° – 25.87°	21.1° – 16.32°	8100 – 4300	±15° – ±22°	<10^−7^	10^−5^ – 10^−6^
6 p.m. – 7 p.m.	25.87 – 37.23°	16.32° – 6.31°	4300 – 2100	±22°	<10^−7^	10^−6^ – <10^−7^
7 p.m. – 7:45 p.m.	37.23° – 45.08°	6.31° – 0.83°	2100 – 980	±22°	<10^−7^	<10^−7^

* Between 10:50 a.m. and 12:45 p.m., the sky was cloudy; ** between 5 p.m. and 5:30 p.m., the sun irradiance suddenly dropped due to a tree that blocked the line of sight between the sun and the VLC receiver.

## Data Availability

Data are contained within the article.
